# Case Report: Severe acute pancreatitis accompanied by gastric mucosal exfoliation hemorrhage: clinical alerts and novel insights

**DOI:** 10.3389/fsurg.2024.1471966

**Published:** 2025-01-28

**Authors:** Yanquan Liu, Hehui Zhang, Minjuan Zeng, Jian Luo, Yun Lai, He Huang, Qinglin Xu, Yuan Liu

**Affiliations:** ^1^ The First School of Clinical Medicine, Guangdong Medical University, Guangdong, China; ^2^ Department of Intensive Medicine (Comprehensive ICU), The First Affiliated Hospital of Gannan Medical University, Ganzhou, China; ^3^ Department of Gastroenterology, The First Affiliated Hospital of Gannan Medical University, Ganzhou, China; ^4^ Department of Dermatology, The First Affiliated Hospital of Gannan Medical University, Ganzhou, China; ^5^ Department of Cardiovascular Medicine (Cardiology), The First Affiliated Hospital of Gannan Medical University, Ganzhou, China; ^6^ Department of Operating Room, The First Affiliated Hospital of Gannan Medical University, Ganzhou, China

**Keywords:** severe acute pancreatitis (SAP), gastric mucosal exfoliation hemorrhage, differential diagnosis, treatment, prognosis

## Abstract

Acute pancreatitis (AP), a condition marked by its swift progression, substantial individual discrepancies, and profound concealment, poses a formidable challenge. Within its severe form, known as severe acute pancreatitis (SAP), the disease assumes an even more prevalent status, often entwined with dire complications such as pancreatic abscess, circulatory shock, and the direst of all, multiple organ failure. Regrettably, the conjunction of SAP with gastric mucosal exfoliation culminating in massive hemorrhage remains an exceptionally rare phenomenon within the clinical realm. This study delves into a retrospective analysis of a singular, yet remarkable clinical case, where SAP's therapeutic odyssey unexpectedly led to gastric mucosa stripping and catastrophic gastrointestinal bleeding. This paper endeavors to unravel the diagnostic intricacies, explore the treatment modalities, and prognosticate the outcome, all with the ultimate aim of fostering a heightened clinical vigilance and fostering a novel, nuanced understanding of SAP's exceptional complications within the intensive care arena. Furthermore, this study aspires to serve as a beacon of reference, illuminating the path for clinical practitioners confronted with such elusive yet critical scenarios.

## Introduction

Acute pancreatitis (AP) is a serious inflammatory disorder marked by the destruction of pancreatic acinar cells. This disease is primarily induced by the abnormal activation of pancreatic enzymes like trypsin, resulting in the self-digestion of pancreatic parenchyma. Based on the Atlanta consensus classification, the severity of AP can be categorized into mild, moderate, and severe using scoring systems such as the Ranson score, BISAP score, APACHE-II score, SOFA score, and so on (1). The global annual incidence of AP ranges from 4.9/100,000 to 73.4/100,000. In China, the incidence has risen from 0.19% to 0.71% over the past 20 years, and approximately one quarter of the patients will develop moderate or severe pancreatitis, with a mortality rate as high as 15% (2, 3). Due to the aging of the population, AP is increasingly prevalent among the elderly. In clinical practice, elderly AP is more severe, with more common systemic complications and a higher mortality rate than young patients (4, 5). Although significant progress has been made in the treatment of AP in recent years, the prognosis of AP remains pessimistic, and its treatment should be comprehensive, where the multidisciplinary linkage treatment involving medicine, surgery, and the ICU is crucial. Therefore, this paper retrospectively investigated the treatment process and outcome of an elderly patient with SAP accompanied by terrifying gastric mucosal exfoliation hemorrhage, analyzed and summarized the successful experience of the diagnosis and treatment of this patient, in order to deepen the understanding of rare complications of SAP in the elderly.

## Clinical presentation

**Figure 1:**
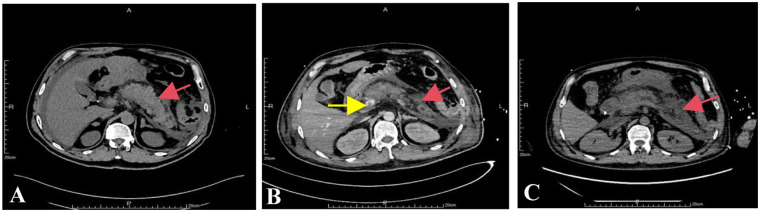
CT examination results of the patient's abdomen. Note: **(A)** CT examination performed in the outpatient clinic; **(B)** whole-abdominal enhanced CT during ICU stay; **(C)** Abdominal CT examination of the second review during ICU stay. The red arrow indicates the gradually expanding necrotic exudation area of the pancreas, and the yellow arrow indicates portal venous occlusion.

A 69-year-old male patient, sought treatment at the First Affiliated Hospital of Gannan Medical University, complaining of “abdominal pain for one day”. The patient experienced abdominal pain after consuming broth before admission to the hospital, characterized by persistent colic in the middle and upper abdomen, more pronounced around the umbilicus, exacerbated after eating. The above symptoms could radiate to both sides of the waist, without nausea or vomiting. The CT scan of the entire abdomen in outpatient department (Figure 1A) indicated: (1) Signs of AP, peritoneal inflammatory changes; (2) Abdominal pelvic effusion, bilateral pleural effusion; (3) Gallbladder duct stones, fatty liver. The patient was admitted to the Department of Gastroenterology of our hospital as “acute biliary pancreatitis”, while the patient's condition failed to improve and even deteriorated progressively, with a poor mental state, chest tightness, shortness of breath, and poor vital signs such as blood oxygen saturation decreased progressively. Thus, the patient was transferred to the ICU, the patient was administered a series of treatments such as norepinephrine pressor therapy, stomach protection, hemostasis, anti-infection, nutritional support, abdominal puncture drainage, and continuous renal replacement therapy (CRRT), the patient still appeared unconscious, shortness of breath, and a decreased blood oxygen saturation of 85%. After high-flow oxygen therapy, the blood oxygen saturation remained lower than 90%, and the vital signs became stable after the immediate establishment of an artificial airway to assist breathing.

**Figure 2:**
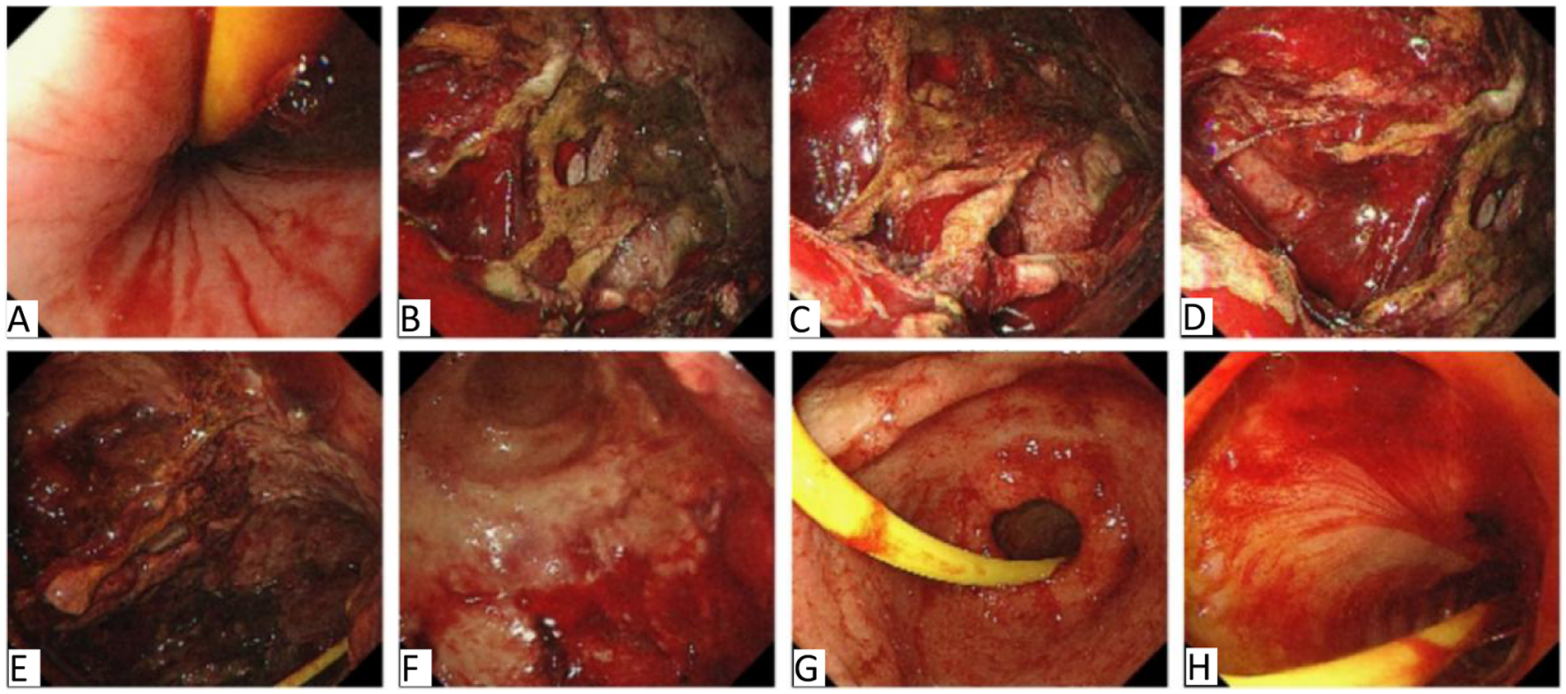
The first gastroscopy results of the patient after admission to ICU. Note: **(A)** Is esophagus, **(B–D)** are cardia of stomach, **(E,F)** are corpus of stomach, **(G)** is antrum of stomach, **(H)** is duodenal bulb segment. Upon entering the esophagus, remnants of blood were distinctly visible. Subsequent suctioning of the area revealed no discernible bleeding points within the esophagus. However, within the stomach cavity, a significant accumulation of blood clots and scabs was encountered. Following a thorough rinse with water, a substantial area of the stomach's cardia and corpus exhibited a pronounced mucosal denudation. To address this, a solution of diluted Adrenaline, mixed with saline, was meticulously sprayed onto the affected regions of the corpus and cardia. Further examination unveiled a minute ulceration in the antrum, characterized by a white coating at its base. Notably, there was no evidence of active bleeding, nor were any bleeding lesions detected in the duodenal bulb or its descending segment.

**Figure 3:**
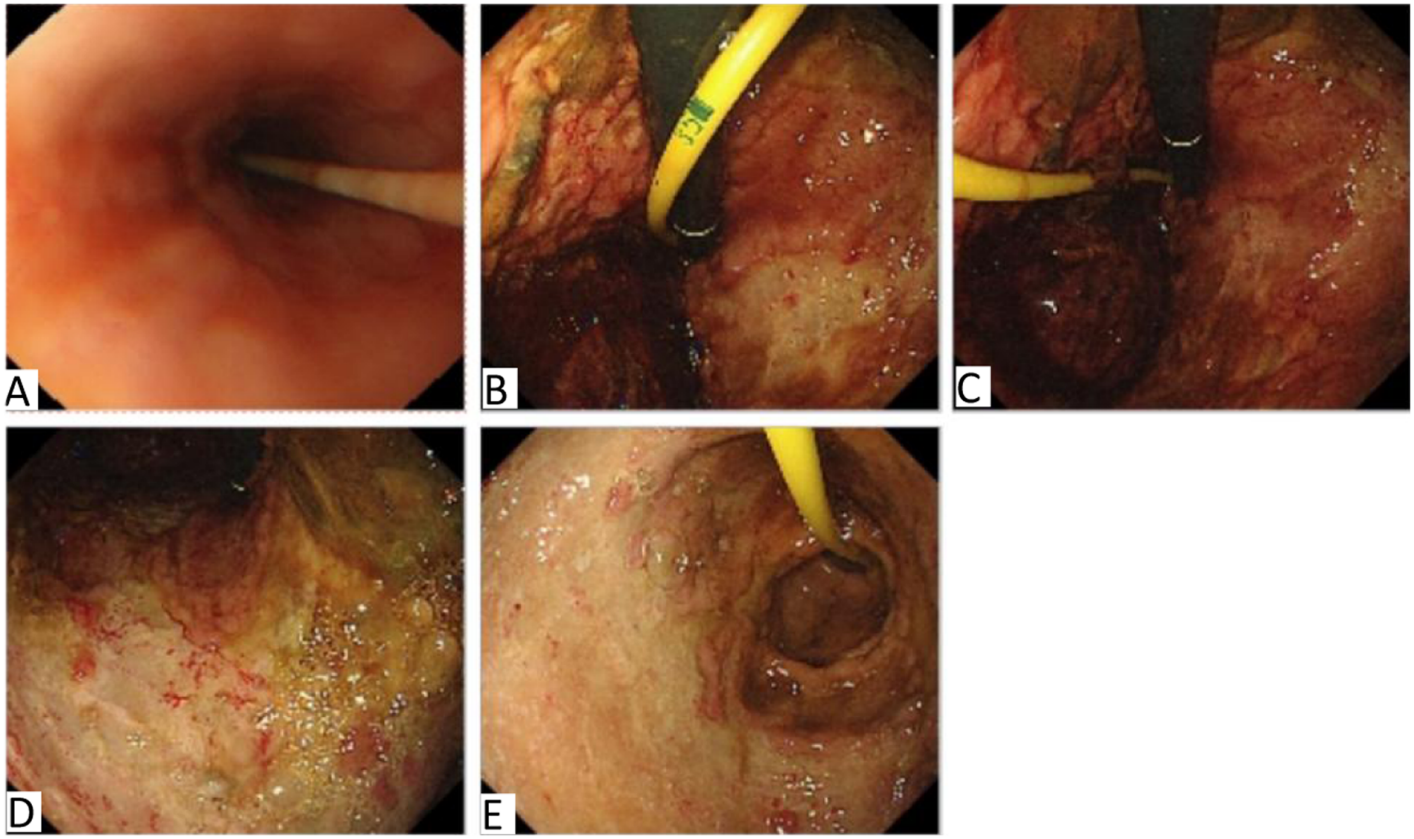
Results of the second gastroscopy after admission to ICU. Note: In this illustration, **(A)** represents the esophagus, **(B)** signifies the gastric fundus, **(C,D)** denotes the gastric body, and **(E)** is the gastric antrum. The esophagus exhibits a smooth and supple mucosa, characterized by a distinct and well-defined blood vessel pattern that indicates excellent distensibility and elasticity. In the gastric fundus, abundant fresh blood and clots suggest hemorrhage. The stomach body, gastric angle and antrum show large mucosal avulsion, mucosal roughness, diffuse bleeding, indicative of significant damage.

During ICU treatment, a whole-abdominal enhanced CT examination (Figure 1B) disclosed signs of acute necrotizing pancreatitis along with accumulation of peripheral necrosis, embolism in the portal vein and superior mesenteric vein, and peritoneal inflammatory alterations. Simultaneously, considering the coagulation dysfunction caused by increased infection, 500 ml of frozen plasma was infused for the patient. Due to the patient manifested a low fever of 37.5°C and was treated with imipenem cilastatin sodium and vancomycin in combination for anti-infective therapy considering bacterial proliferation and infection in the abdominal cavity. Moreover, our treatment team believed that off-line extubation could not be carried out in the short term, so tracheotomy and ventilator-assisted breathing were conducted for the patient. Abdominal CT examination of the patient a few days later (Figure 1C) showed signs of acute necrotizing pancreatitis with accumulation of surrounding necrosis and increased necrosis. The embolization of the portal vein and superior mesenteric vein was similar to the previous condition. Peritoneal inflammatory changes were improved compared to before. On the 6th day after the patient was admitted to the ICU, a state of balance was achieved between the intake and output volumes of patient's urine. Consequently, the CRRT was successfully terminated. Furthermore, the patient's physical manifestations and inflammatory markers displayed notable improvement, and vital signs remained consistently stable. To expedite the recovery of the patient's digestive functions, an adequate regimen of enteral nutrition was administered. On the 9th day of ICU admission, brown-hued drainage fluid was observed in the patient's gastrointestinal decompression apparatus, the enteral nutrition was temporarily suspended. Subsequent reexamination of coagulation analysis and blood routine indicated moderate anemia and abnormal coagulation, and transfusion support was immediately given. Meanwhile, considering that the blood gas analysis indexes were still unstable after the removal of ventilator support, oxygen was administered through nasal catheter after tracheotomy, and low-dose sedation and analgesia measures were given. On the 13th day after the patient was admitted to the ICU, he endured a severe episode of hematemesis accompanied by substantial blood clots, following the electronic gastroscopy examination (Figure 2), a notable detachment of a substantial mucosal layer was discerned in the fundus and body regions of the stomach. Given the intractability of endoscopic intervention for this specific case of gastric mucosal detachment, a course of conservative therapy was initiated, and it included inhibition of enzymatic activity, arrest bleeding, protection of gastric mucosa and other means. Concurrently, the patient's blood oxygen levels remained erratic, prompting the continuation of tracheotomy and the utilization of a ventilator to provide crucial respiratory support. Given the patient's condition of anemia coupled with inadequate blood coagulation capabilities, a comprehensive transfusion therapy was administered. The following day, the patient abruptly manifested delirium and was unresponsive to verbal stimuli. To identify the underlying cause, a brain MRI scan was conducted, yet the results indicated no significant anomalies. Subsequently, we conducted a second follow-up gastroscopy on the patient (Figure 3), and discovered large mucosal detachment, diffuse bleeding in the main body of the stomach, the angle of the stomach and the antrum, and mucosal irregularization. The overall condition of the patient was significantly improved after receiving treatment measures such as reducing enzyme, protecting stomach, inhibiting acid, hemostasis and antiemesis. After 23 days of ICU treatment and intensive nursing, the patient's condition further improved, vital signs were stable, and he was transferred out of ICU. Up to now, it revealed that the patient's quality of life, daily functioning, and mental health were favorable, with no remarkable long-term adverse sequelae.

## Discussion

Acute pancreatitis (AP) stands as a prevalent and potentially life-threatening condition affecting the digestive system. This ailment arises from the devastation of pancreatic acinar cells coupled with the abnormal activation of pivotal enzymes, notably trypsin, which triggers the autoregressive digestion of pancreatic parenchyma. Consequently, patients frequently present to medical facilities with vague symptoms like abdominal discomfort and nausea (6, 7), while elderly individuals may experience diagnostic oversights due to non-canonical abdominal pain manifestations. The underlying pathological process of AP primarily stems from the self-destructive nature of pancreatic enzymes upon the pancreas itself and its adjacent tissues, precipitating a cascade of organ dysfunctions. Intriguingly, pancreatogenic upper gastrointestinal bleeding, though an uncommon clinical entity, poses a grave threat to life in cases of profuse hemorrhage. Thus, enhancing our comprehension of this rare yet perilous form of gastrointestinal hemorrhage—pancreatogenic in origin—is paramount.

AP can be diagnosed through a combination of clinical symptoms such as continuous upper abdominal pain, amylase levels, and imaging techniques. In this case, the patient had a history of abdominal pain when referred to our hospital, with a normal serum amylase level and typical imaging manifestations of pancreatitis, which meet the diagnostic criteria for AP. In addition, the patient's organ dysfunction (creatinine value greater than 300 μmol/L) lasted for more than 48 h. According to the revised Atlanta classification (RAC), the patient can be diagnosed with severe AP (1). During the treatment of AP, endoscopy is an important diagnostic and therapeutic tool. The timing of endoscopy should be determined based on the specific condition and treatment progress of the patient. Generally speaking, if the patient presents with symptoms such as hematemesis and melena, endoscopy should be performed immediately to determine the location and cause of bleeding, and timely treatment should be provided. If the patient exhibits hemodynamic instability, active fluid resuscitation and blood transfusion therapy should be given firstly, and emergency endoscopy should be arranged immediately after hemodynamic stability is corrected (8). The condition of gastric mucosal denudation is known to manifest itself in a multitude of discomforting symptoms, ranging from abdominal pain and nausea to vomiting and, notably, gastrointestinal bleeding, as has been previously reported (9). In this specific case, the patient unfortunately underwent a traumatic experience of upper gastrointestinal bleeding during the progression of their medical condition. The endoscopic evaluation, conducted subsequently, painted a vivid picture of the severity of the situation, revealing a substantial and vast expanse of gastric mucosal denudation, thereby highlighting the urgency and importance of addressing this issue promptly.

AP frequently incurs harm upon the upper gastrointestinal mucosa, with the degree of damage intensifying as the severity of AP escalates (10). It is imperative to discern it from hemorrhagic necrotizing gastritis, and abdominal CT serves as a valuable tool in differentiating between these two pathologies. In the case of necrotizing gastritis, the foci predominantly reside within the gastric antrum, manifesting as diffuse thickening of the antral wall and a layered appearance within the thickened gastric wall. For pancreatitis-associated gastrointestinal bleeding, CT scans can reveal the accumulation of necrotic debris surrounding the pancreas. Chen et al. conducted a study and observed that among 197 patients with AP, 128 patients (65%) exhibited gastrointestinal mucosal lesions identified through endoscopy, indicating a potential benefit from acid suppression therapy (11). Additionally, several studies have highlighted a relatively high incidence of peptic ulcer in AP patients, reaching 52.6% (12). Nevertheless, there is a paucity of specific data regarding the prevalence of gastric mucosal detachment stemming from SAP. The academic fraternity posits that the etiology and mechanisms underlying gastric mucosal damage in AP may stem from a cascade of inflammatory reactions mediated by inflammatory mediators generated by macrophages, ultimately leading to gastric mucosal injury and dysfunction. Notably, the extent of gastric mucosal damage tends to be more pronounced in elderly patients (13, 14).

Furthermore, in patients suffering from SAP, particularly those who necessitate intensive care therapy or mechanical ventilation, the likelihood of gastric mucosal damage escalates significantly due to stress factors (15). Stress elicits the release of gastrin and gastric acid, with the subsequent infiltration of gastric acid into the mucosa precipitating mucosal injury. A comprehensive literature review further underscores instances where splenic venous thrombosis, a known complication of AP, gives rise to gastric varices, which in turn, can cause bleeding. This complication arises from thrombosis impeding splenic venous outflow, rerouting blood flow through the gastric short vein, submucosa, and perigastric branches into the portal vein via the left gastric vein, fostering the development of gastric varices (16). While in our study, the patient received prolonged infusions of high-dose noradrenaline to stabilize blood pressure upon admission. Consequently, the potential for vasoactive drug-induced ischemia of minute gastric vessels and subsequent mucosal detachment cannot be overlooked, necessitating clinical vigilance and differential diagnosis. Although the patient was promptly treated with acid-suppressing medications upon ICU admission, vomiting of blood manifested on the 14th day of illness, revealing gastric mucosal detachment. Swift interventions including acid suppression, gastric protection measures, blood transfusions, and the administration of noradrenaline + ice-saline solution through a gastric tube mitigated the symptoms of gastrointestinal bleeding. Intriguingly, despite the prevalent use of acid-suppressing agents in managing pancreatitis cases, current guidelines for pancreatitis management do not explicitly address the administration of these drugs (17). This observation underscores the need for further research and updates to existing guidelines to ensure optimal patient care.

In-depth research has uncovered that when comparing patients with SAP undergoing standard treatment with those additionally administered proton pump inhibitors (PPIs), no discernible therapeutic advantage emerges in mitigating systemic inflammatory responses or enhancing clinical outcomes, and PPIs fail to thwart the onset of peptic ulcers and gastrointestinal bleeding (18). It is crucial to emphasize that, despite their inefficacy in preventing gastrointestinal bleeding among severe pancreatitis patients, acid-suppressing agents hold paramount significance in treating individuals who have developed gastrointestinal mucosal lesions due to pancreatitis. On the positive side, by diminishing gastric acidity, these agents mitigate its irritant effect on the gastric mucosa, thereby contributing to the prevention or treatment of peptic ulcers. Furthermore, they curtail the secretion of pancreatic juice, ultimately alleviating pancreatic duct hypertension (19). This multifaceted approach underscores the strategic importance of employing acid-suppressing agents in the management of pancreatitis patients with gastrointestinal complications.

For the treatment of AP and SAP, enteral nutrition (EN) is the preferred form of nutritional support for SAP patients as it can maintain intestinal barrier function and reduce infectious complications. Early enteral nutrition (EEN) should be initiated within 24–48 h after admission to prevent intestinal barrier dysfunction and infectious complications. Parenteral nutrition (PN) is indispensable for patients who cannot tolerate enteral nutrition (20, 21). Additionally, pain management is particularly crucial, and the utilization of opioids should be prioritized for pain control, but the specific drug selection should be determined by the patient and the doctor (22). For patients with infectious pancreatic necrosis, antibiotics known to penetrate pancreatic necrosis are recommended; prophylactic antibiotics are not advocated for patients with aseptic necrosis (23). Simultaneously, fluid resuscitation is especially essential in clinical practice, and moderate aggressive fluid resuscitation is recommended in the initial treatment of SAP patients to optimize tissue perfusion. In the acute phase of SAP, systematic massive fluid resuscitation (3–5 ml/kg/h) is not recommended to decrease mortality, acute renal failure, or the length of hospital stay (23). Of course, in clinical practice, some AP or SAP patients will continue to deteriorate and have multiple organ dysfunction, and they should be considered for admission to the ICU for close monitoring and supportive treatment, as well as close imaging evaluation (21, 23). For patients with SAP, contrast-enhanced CT (CE-CT) or MRI evaluation is recommended within 72–96 h after the onset of symptoms. For infectious pancreatic necrosis, surgical intervention is recommended at the appropriate time to reduce the risk of surgery and enhance the therapeutic effect. Especially in SAP patients, if intra-abdominal hypertension (IAH) or intra-abdominal syndrome (ACS) is present, open abdominal surgery may be necessary to alleviate symptoms (21–23). Of course, for SAP due to specific etiologies, such as SAP due to hyperlipidemia, therapeutic plasma exchange (TPE) can be contemplated to rapidly reduce severe hypertriglyceridemia if drug therapy fails (21).

It should be noted that secondary infection of necrotic tissue in acute necrotizing pancreatitis (ANP) is not infrequent in clinical practice and is associated with a high mortality rate. The step-up approach (SUA) and open necrotomy (ON) are reliable and invasive treatments for ANP (24). In the treatment of ANP combined with infectious pancreatic necrosis, ON is the traditional approach to achieve adequate source control and removal of infected necrotic tissue. However, in recent years, in order to improve the survival rate of ANP patients while focusing on reducing complications, minimally invasive techniques such as percutaneous drainage (transabdominal or retroperitoneal), transgastric drainage, and video-assisted retroperitoneal debridement (VARD) have been developed (3, 25). It also indicated that antibiotics and supportive treatment are optimal for suspected infectious necrosis (26). In cases of persistent sepsis, retroperitoneal percutaneous drainage is the first step, and a minimally invasive retroperitoneal necrosis resection route is considered if necessary. If there is no clinical improvement after 72 h, a second drainage operation or full drainage with the VARD minimally invasive technique should be performed.

## Conclusion

In conclusion, the reasons that lead to the erosion of the gastric mucosa in patients suffering from AP are multifarious, encompassing the liberation of inflammatory mediators, heightened neural excitability, impaired gastric mucosal barrier functionality, and potentially, the prolonged administration of high-dose vasoactive medications. In the realm of clinical practice, instances of gastrointestinal bleeding stemming from pancreatitis necessitate utmost attention. Particularly in patients with SAP, coupled with stressors such as hypoxia, mechanical ventilation, utilization of non-steroidal anti-inflammatory drugs, or inadequate gastrointestinal perfusion, we must maintain heightened vigilance towards the potential for hemorrhage. Prompt enhancement of gastroscopy examinations is paramount to elucidate the underlying cause of bleeding, and swift implementation of early gastrointestinal decompression, fluid resuscitation, pancreatic secretion suppression, and gastric mucosal protective therapy is indispensable. For patients who remain undiagnosed or are inadequately treated, a multi-pronged approach incorporating diverse diagnostic and therapeutic modalities can significantly enhance the rate of successful diagnosis and treatment outcomes.

## Data Availability

The original contributions presented in the study are included in the article/Supplementary Material, further inquiries can be directed to the corresponding author.
